# *MYSM1* regulates the proliferation and differentiation of bovine skeletal muscle satellite cells via *BRG1*-mediated activation of the *AKT/mTOR/NF-κB* signaling pathway

**DOI:** 10.5713/ab.250773

**Published:** 2025-12-18

**Authors:** Chujie Zhang, Yue Li, Wenwen Zhang, Tengxia Ma, Xin Li, Yiwen Guo, Linlin Zhang, Xiangbin Ding, Debao Hu

**Affiliations:** 1 Key Laboratory of Animal Breeding and Healthy Livestock Farming, College of Animal Science and Veterinary Medicine, Tianjin Agricultural University, Tianjin, China

**Keywords:** *AKT*, *mTOR*, *NF-κB* Signaling Pathway, Bovine Skeletal Muscle Satellite Cells, *BRG1*, Epigenetic Modifications of Histones, *MYSM1*

## Abstract

**Objective:**

This study investigates the molecular mechanisms by which the deubiquitinase *MYSM1* regulates the proliferation and differentiation of bovine skeletal muscle satellite cells (BSMSCs), thereby providing new theoretical insights into the regulation of muscle growth and development in beef cattle.

**Methods:**

An *in vitro* model of BSMSCs was established to investigate the role of *MYSM1*. The expression pattern of *MYSM1* during cell proliferation and differentiation was analyzed using quantitative real-time polymerase chain reaction (qRT-PCR). *MYSM1* knockdown models were generated, and the expression of proliferation markers *PAX7* and *Ki67*, as well as differentiation markers *MYHC* and *MYOG*, were examined by qRT-PCR and Western blotting. Analysis of protein–protein interactions identified *BRG1* as a potential *MYSM1* interactor, and its function was subsequently evaluated. Downstream signaling activity was evaluated by examining phosphorylation changes in key components of the AKT/mTOR pathway. In addition, global histone ubiquitination *H2AK119ub1* and the histone methylation markers *H3K4me3* and *H3K27me3* were analyzed following *MYSM1* knockdown.

**Results:**

*MYSM1* expression was dynamically regulated, exhibiting significant upregulation during differentiation, reaching its highest at days 2–3 (p<0.05). Silencing of *MYSM1* significantly (p<0.05) decreased the expression of *PAX7*, *Ki67*, *MYHC*, and *MYOG*. Histone modification analyses demonstrated elevated levels of *H2AK119ub1* and *H3K27me3*, along with reduced *H3K4me3* (p<0.05). Mechanistic studies showed that MYSM1 knockdown significantly reduced *BRG1* expression (p<0.05), and *BRG1* silencing similarly decreased proliferation and differentiation markers. Moreover, interference with either *MYSM1* or *BRG1* significantly attenuated the activation of the *AKT/mTOR/NF-κB* signaling pathway, as evidenced by decreased phosphorylation of *AKT*, *mTOR*, and *p65* (p<0.05).

**Conclusion:**

*MYSM1* promotes the proliferation and differentiation of BSMSCs through *BRG1*-mediated epigenetic regulation and activation of the *AKT/mTOR/NF-κB* signaling cascade. These findings establish a dual-target framework that advances the understanding of muscle development in beef cattle and offers potential strategies for regenerative therapies.

## INTRODUCTION

Skeletal muscle is an essential tissue involved in exercise and metabolism and also represents a major component of meat products in livestock and poultry. The growth and development of skeletal muscle largely determine meat yield and quality, thereby exerting a substantial impact on the economic efficiency of modern animal husbandry [1]. Owing to its strong regenerative capacity, skeletal muscle can effectively respond to mechanical stress and injury. Bovine skeletal muscle satellite cells (BSMSCs), a population of tissue-specific stem cells located beneath the muscle fiber basement membrane, play a central role in muscle repair and regeneration by undergoing proliferation and differentiation, thereby serving as key regulators of muscle homeostasis [2]. Increasing evidence indicates that skeletal muscle differentiation is governed not only by genetic factors but also by diverse epigenetic mechanisms, including histone modifications, spatiotemporally regulated assembly of transcription factor complexes, and three-dimensional chromatin reorganization. Together, these mechanisms orchestrate the fate determination and precise differentiation of skeletal muscle precursor cells [3].

*Histone H2A lysine 119 monoubiquitination* (*H2AK119ub1*) is a well-established marker of transcriptional repression and plays a pivotal regulatory role in inhibiting transcription, cell cycle progression, and DNA damage repair [4]*.* The *Myb-like, SWIRM* and *MPN domains 1* (*MYSM1*) gene encodes a specific deubiquitinase responsible for removing *H2AK119ub1*, thereby exerting profound effects on stem cell function, immune regulation, and the pathogenesis of various diseases. It is essential for maintaining stem cell homeostasis. Functional loss of *MYSM1* has been associated with pathological conditions such as lymphopenia, anemia, and thrombocytosis [5]. Moreover, *MYSM1* deficiency drives hematopoietic stem cells (HSCs) out of quiescence and into rapid cycling, ultimately accelerating their apoptosis [6]. In *MYSM1*-deficient mice, the number of T lymphocyte precursors is markedly reduced compared with wild-type controls, and CD8-positive T cells (CD8^+^ T cells) exhibit hyperactivation followed by a sharp decline in cell number [7]. *MYSM1* also exerts critical effects on dendritic cells (DCs), as knockout studies have revealed a dramatic reduction in DC numbers within the lymphatic system [8]. Interestingly, in models of systemic lupus erythematosus, *MYSM1* deficiency results in an abnormal expansion of B1-a cells [9]. Furthermore, *MYSM1* knockout mice exhibit markedly reduced bone mass in the skull and long bones, despite no detectable changes in osteoblast precursor proliferation, whereas the adipogenic capacity of bone marrow mesenchymal stem cells (MSCs) is significantly enhanced [10]. Mechanistically, *MYSM1* promotes chromatin accessibility by deubiquitinating *H2AK119ub1*, thereby facilitating transcriptional activation [11]. Although *MYSM1* has been shown to exert critical regulatory functions across multiple stem cell systems, its molecular role in muscle stem cells remains largely unexplored. *Brahma-related gene 1* (*BRG1*) also known as *SMARCA4*, is one of the two catalytic subunits of the sucrose non-Fermentable chromatin-remodeling complex (SWI/SNF) ATP-dependent chromatin remodeling complex, which plays a central role in DNA replication, repair, recombination, and transcription. Accumulating evidence indicates that *BRG1* participates in the regulation of multiple cell cycle processes associated with redox signaling [12]. Moreover, numerous studies have shown that *BRG1* exerts distinct biological functions in different cellular contexts. In recent years, studies have shown that *BRG1* displays strikingly context-dependent functions in cancer, acting as either a tumor promoter or a tumor suppressor depending on the signaling pathways it interacts with. Because *BRG1* engages distinct regulatory networks in different cellular environments, its effects on proliferation or apoptosis vary substantially among cancer types [13]. In addition, other studies have shown that in lung cancer, *BRG1* has been reported to inhibit cell proliferation and metastasis by inactivating the *Wnt/β-catenin* signaling pathway [14]. In addition, in a mouse HDM-induced asthma model, *BRG1* expression is elevated in airway epithelial cells and interacts with *phosphoinositide 3-kinase* (*PI3K*). Genetic deletion of *BRG1* unexpectedly enhances phosphoinositide *PI3K/protein kinase B* (*AKT*)*/mechanistic target of rapamycin* (*mTOR*) signaling pathway and alleviates airway inflammation, and this protective effect is abolished by the *PI3K* inhibitor LY294002. These findings indicate that *BRG1* exacerbates airway inflammation by suppressing *PI3K/Akt/mTOR* pathway activity [15]. At the same time, *BRG1*-mediated chromatin remodeling is indispensable not only for skeletal muscle differentiation but also for maintaining the pool of myoblasts [16].

The *AKT/mTOR/nuclear factor kappa-light-chain-enhancer of activated B cells* (*NF-κB*) signaling pathway plays a pivotal role in determining cell fate by orchestrating cell growth, proliferation, and cell cycle regulation. Recent studies have shown that *MSTN* (*Myostatin*) regulates the differentiation of BSMSCs through a *proteasome subunit alpha type-6* (*PSMA6*)*-*mediated activation of the *AKT* signaling pathway. This evidence underscores the essential role of *AKT* signaling in driving myogenic progression in cattle [17]. Concurrently, age-related activation of *NF-κB* signaling in myofibers disrupts the satellite cell niche and diminishes their regenerative potential. These findings indicate that chronic *NF-κB* activity contributes significantly to the decline in muscle repair capacity during aging, highlighting the pivotal role of this pathway in regulating satellite cell function and skeletal muscle homeostasis [18]. Moreover, *MYSM1* acts as a negative regulator of innate immune signaling by deubiquitinating *TNF receptor-associated factor 3* (*TRAF3*) and *TNF receptor-associated factor 6* (*TRAF6*), thereby limiting *NF-κB* and type I interferon activation and preventing excessive inflammation [19]. Accordingly, the present study aimed to elucidate the regulatory function of *MYSM1* in the proliferation and differentiation of BSMSCs and to uncover the molecular mechanism whereby *MYSM1* activates the target gene *BRG1* to modulate the *AKT/mTOR/NF-κB* signaling cascade. These findings are expected to fill the current knowledge gap regarding the role of *MYSM1* in skeletal muscle stem cells and to provide a novel molecular target for enhancing the quality and efficiency of muscle development in livestock and poultry.

## MATERIALS AND METHODS

### Cell isolation and culture with Dulbecco’s modified Eagle’s medium

In this study, all cells were isolated, cryopreserved, and provided by the Key Laboratory of Agricultural Animal Breeding and Healthy Aquaculture, Tianjin. First, the cells preserved in liquid nitrogen were thawed and transferred into T25 flasks for culture. The cells were maintained in proliferation medium until reaching approximately 90% confluence, after which they were passaged for subsequent use. Proliferating cells were maintained in modified Dulbecco’s modified Eagle’s medium (DMEM, high glucose; HyClone) supplemented with 20% fetal bovine serum (FBS; Gibco, USA) and 1% penicillin–streptomycin solution (Solarbio). For differentiation, cells were cultured in DMEM containing 2% horse serum (HS; Gibco) and 1% penicillin–streptomycin solution.

### Synthesis of si-RNA

All siRNAs used for gene knockdown, along with the negative control (siNC), were synthesized by Ruibo Biotech, and their sequences are listed in Tables 1, 2.

### Cell transfection

BSMSCs were seeded into 6-well and 12-well plates and transfected when cell density reached approximately 70%, following the manufacturer’s protocol for Lipofectamine 3000 (Invitrogen). Three biological replicates were established for both the experimental and control groups. Cells were harvested at 24, 48, and 72 hours post-transfection for subsequent analyses.

### Quantitative real-time polymerase chain reaction analysis

Total RNA was extracted using TRIzol reagent (Invitrogen) Briefly, cells were lysed thoroughly in TRIzol, and the aqueous phase was separated by chloroform centrifugation (12,000×g, 15 min, 4°C). RNA was precipitated with isopropanol, washed twice with 75% ethanol, air-dried, and dissolved in RNase-free water. RNA concentration and purity were assessed using a NanoDrop 2000 spectrophotometer (Thermo Fisher Scientific), and samples with A260/280 ratios between 1.9 and 2.1 were used for subsequent experiments. RNA integrity was verified by 1% agarose gel electrophoresis, and cDNA was synthesized with the PrimeScript cDNA Synthesis Kit (Takara). The reverse transcription procedure was carried out according to the manufacturer’s protocol: 42°C for 2 min (gDNA removal), followed by 37°C for 15 min and 85°C for 5 s to inactivate reverse transcriptase. Quantitative real-time polymerase chain reaction (qRT-PCR) was performed using the All-in-One qPCR Mix (Genecopoeia) on a LightCycler 96 system (Roche). Each 20 μL reaction contained 10 μL of 2× qPCR Master Mix, 0.4 μL of each primer (10 μM), 2 μL of diluted cDNA template, and RNase-free water to volume. The amplification protocol was as follows: initial denaturation at 95°C for 10 min, followed by 40 cycles of 95°C for 10 s and 60°C for 30 s. Melt curve analysis (65°C–95°C, 0.5°C increments) was performed to confirm primer specificity. The primer sequences used for amplification are listed in Table 3.

### Western blot analysis

Total protein was extracted from undifferentiated BSMSCs and differentiated myotubes using RIPA buffer (Takara), Cell lysates were incubated on ice for 10 min and then centrifuged at 12,000×g for 15 min at 4°C to remove insoluble material. The supernatant was collected, and protein concentration was determined with a NanoDrop 2000c spectrophotometer (Thermo Fisher Scientific). Equal amounts of protein (40 μg per sample) were mixed with 5× loading buffer, boiled at 95°C for 5 min, and resolved by SDS–PAGE on 8%–12% polyacrylamide gels depending on the molecular weight of the target proteins. Proteins were subsequently transferred onto PVDF membranes (Millipore) using a wet transfer system. After transfer, membranes were briefly rinsed in TBST (20 mM Tris-HCl, 150 mM NaCl, 0.1% Tween-20, pH 7.4) and then blocked with 5% (w/v) nonfat dry milk in TBST for 2 h at room temperature. Blocked membranes were incubated overnight at 4°C with the appropriate primary antibodies diluted in blocking buffer. After three washes with TBST (10 min each), membranes were incubated with HRP-conjugated secondary antibodies for 1–2 h at room temperature. Following three additional washes in TBST, immunoreactive bands were visualized using an enhanced chemiluminescence (ECL) detection kit (Boster) and imaged with a chemiluminescence imaging system. Membranes were incubated with primary antibodies against *MYSM1* (1:1,000; Abcam), *myogenin* (*MYOG*) (1:100; DSHB), *myosin heavy chain* (*MYHC*) (1:100; DSHB), *paired box 7* (*PAX7*) (1:500; Sangon Biotech), *glyceraldehyde-3-phosphate dehydrogenase* (*GAPDH*) (1:5,000; Abmart), *tubulin* (1:100; Bioss), *AKT1* (1:1,000; Abmart), *p-AKT1* (Ser473, 1:1,000; Abmart), *mTOR* (1:1,000; Abmart), *p-mTOR* (1:1,000; Bioss), *p65* (1:5,000; Abmart), *p-p65* (1:1,000; Abmart), *H2AK119ub1* (1:1,000; Abcam), *histone H3 lysine 4 trimethylation* (*H3K4me3*) (1:1,000; Abmart), and *histone H3 lysine 27 trimethylation* (*H3K27me3*) (1:1,000; Abmart). All procedures were performed according to the manufacturers’ instructions. The downstream target genes of *MYSM1* were predicted, and the interactions between the downstream target gene *BRG1* and key proteins in the *AKT/mTOR* pathway were predicted. Band intensities were quantified using ImageJ software. The expression levels of target proteins were normalized to GAPDH or β-actin as internal loading controls. All Western blot experiments were performed with at least three independent biological replicates.

### 5-Ethynyl-2′-deoxyuridine

Cell proliferation was assessed using an 5-ethynyl-2′-deoxyuridine (EdU) incorporation assay. BSMSCs were transfected with siRNA, and EdU labeling was performed 24 h after interference. The assay was conducted using a commercial EdU Apollo staining kit (Beyotime Biotechnology), following the manufacturer’s instructions. Briefly, cells were incubated with 200 μM EdU working solution for 3 h at 37°C, fixed with 4% paraformaldehyde for 30 min, and permeabilized with 0.5% Triton X-100 for 10 min. The Apollo reaction cocktail was then applied to visualize EdU-positive nuclei, followed by Hoechst 33342 staining to label total nuclei. Fluorescent images were captured using a fluorescence microscope, and the EdU-positive rate was calculated as the proportion of EdU-labeled nuclei relative to the total number of nuclei. Three biological replicates were included for each group (n = 3 per group).

### Statistical analysis

All experiments were independently repeated at least three times. Data are presented as mean±standard deviation (SD). Statistical analysis was performed using SPSS software (ver. 17.0; IBM). For comparisons between two groups, Student’s t-test was applied. For multiple group comparisons, one-way analysis of variance (ANOVA) was used, followed by Dunnett’s post hoc test for comparison with the control group. Differences were considered statistically significant at * p<0.05 or ** p<0.01, as indicated in the s. All graphs were generated using GraphPad Prism 8 (GraphPad Software).

### Schematic diagram preparation

The mechanistic diagram was generated using BioRender ( https://www.biorender.com ). The illustration was constructed strictly on the basis of the experimental results and the interpretations provided in the Results and Discussion sections, ensuring that the graphical representation accurately reflects the findings of this study.

## RESULTS

### Effects of *MYSM1* on the proliferation and differentiation of bovine skeletal muscle satellite cells

**Figure 1:**
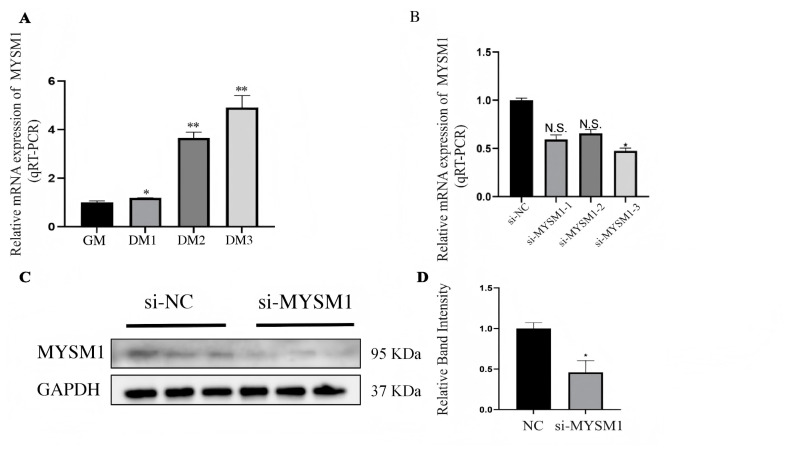
The expression level of *MYSM1* in the proliferation and differentiation process of BSMSCs and screening of interfering sequences. (A) qRT-PCR detection of changes in *MYSM1* expression levels during proliferation and differentiation on the first, second, and third days. GM denotes cell proliferation period and DM for cell differentiation period. (B) The expression level of *MYSM1* was detected via qRT-PCR 24 hours after transfection. (C) Western blotting was used to detect the protein expression levels of *MYSM1* and *GAPDH*. (D) Western blot band quantification is expressed as the ratio of *MYSM1*/*GAPDH* band intensity. Data are presented as mean±SD. * p<0.05, ** p<0.01 vs. control group (one-way ANOVA followed by Dunnett’s test). qRT-PCR, quantitative real-time polymerase chain reaction; BSMSC, bovine skeletal muscle satellite cell; SD, standard deviation; ANOVA, analysis of variance.

To investigate the dynamic regulatory characteristics of the *MYSM1* gene during BSMSC proliferation and differentiation, we analyzed its temporal expression profile across both processes. The results showed that following the induction of differentiation, *MYSM1* mRNA levels increased significantly (p<0.05), with a pronounced elevation on day 2 (D2) and day 3 (D3). At these time points, *MYSM1* expression was markedly higher than that observed during the proliferative phase (p<0.01) (Figure 1A). This differentiation-dependent upregulation of *MYSM1* suggests that it may play an essential role in regulating the proliferation and differentiation of BSMSCs. To further clarify the functional role of *MYSM1*, three specific small interfering RNAs *(si-MYSM1-1*, *si-MYSM1-2*, *and si-MYSM1-3*) were designed to silence its expression. As shown in Figure 1B, *si-MYSM1-3* exhibited the strongest knockdown efficiency compared with the control group, significantly reducing both the mRNA and protein levels of *MYSM1* (p< 0.05) (Figures 1C, 1D). Therefore, *si-MYSM1-3* was selected for subsequent functional analyses due to its superior silencing efficiency.

**Figure 2:**
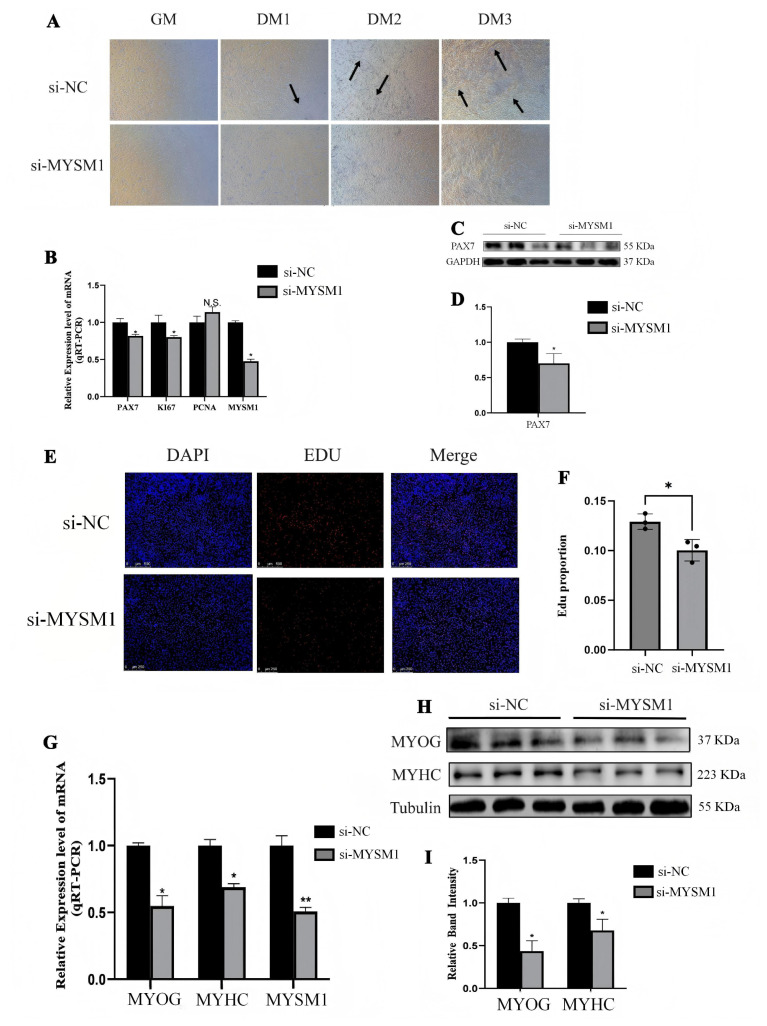
*MYSM1* promotes cell proliferation and differentiation. (A) In the *MYSM1* knockdown experiment, optical microscopy revealed that *MYSM1* silencing resulted in a reduced number of myotubes and decreased myotube size (as indicated by the arrows) (scale bar = 100 μm). GM denotes the cell proliferation phase, and DM denotes the cell differentiation phase. (B) *MYSM1* knock down resulted in a significant decrease in the mRNA expression of the cell proliferation-related markers *PAX7* and *Ki67*. (C) Western blotting was used to detect the protein expression levels of *PAX7* and *GAPDH*. (D) Western blot band determination. (E, F) EdU assay showed that the number of proliferating cells decreased after *MYSM1* gene interference. The knockdown of (G–I) *MYSM1* led to a decrease in the mRNA and protein expression levels of the cell differentiation related markers *MYOG* and *MYHC*. Data are presented as mean±SD. * p<0.05, ** p<0.01 vs. control group (one-way ANOVA followed by Dunnett’s test). qRT-PCR, quantitative real-time polymerase chain reaction; EdU, 5-ethynyl-2′-deoxyuridine; SD, standard deviation; ANOVA, analysis of variance.

To systematically investigate the regulatory role of *MYSM1* in the proliferation and differentiation of BSMSCs, loss-of-function experiments were conducted using the *si-MYSM1* interference model. Morphological observations showed that *MYSM1* knockdown markedly inhibited myotube formation compared with the control group (Figure 2A). Dynamic analysis of proliferation-related markers further revealed that inhibition of *MYSM1* significantly decreased the expression levels of the proliferation markers *PAX7* and *Ki67* (p<0.05) (Figures 2B–2F). Furthermore, analysis of differentiation markers showed that *MYSM1* knockdown significantly reduced both the mRNA and protein expression levels of the differentiation-related factors *MYHC* and *MYOG* (p<0.05), consistent with the observed morphological changes (Figures 2G–2I). Collectively, these findings indicate that *MYSM1* positively regulates the proliferation and differentiation of BSMSCs by promoting the expression of key genes involved in these processes. Loss of *MYSM1* function markedly suppresses both the proliferative capacity and the myogenic differentiation potential of BSMSCs.

**Figure 3:**
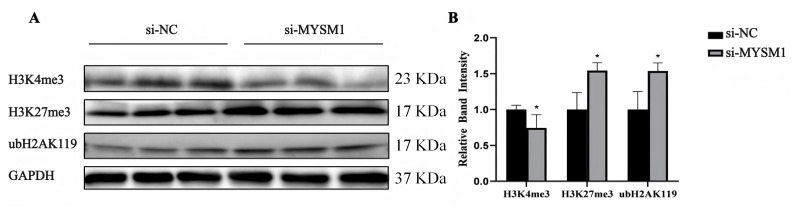
Knocking down *MYSM1* altered intracellular histone modifications. (A) Epigenetic modifications of *H3K4me3/H3K27me3/ubH2AK119* histones were examined by using Western blot. (B) Then, the band quantification was expressed as the ratio of *H3K4me3/H3K27me3/ubH2AK119/GAPDH* band intensity. Data are presented as mean±SD. * p<0.05 vs. control group (one-way ANOVA followed by Dunnett’s test). SD, standard deviation; ANOVA, analysis of variance.

To elucidate the biological functions mediated by *MYSM1*, we systematically examined the dynamic changes in key histone modifications. Compared with the control group, MYSM1 knockdown markedly increased the level of its substrate *H2AK119ub1* (p<0.05), confirming that *MYSM1* contributes to the maintenance of chromatin homeostasis by catalyzing H2A deubiquitination (Figure 3A). Further analysis revealed that the histone methylation landscape was also remodeled: the level of the transcriptional activation marker *H3K4me3* was significantly decreased (p<0.05), whereas the level of the transcriptional repression marker *H3K27me3* was significantly increased (p<0.05) (Figures 3A, 3B). Mechanistic correlation analysis indicated that this histone modification pattern—characterized by the accumulation of *H2AK119ub1* and the reciprocal changes in *H3K4me3* and *H3K27me3*—may enhance chromatin compaction and shift the epigenetic balance toward transcriptional repression. Such alterations are likely to result in the transcriptional silencing of proliferation- and differentiation-related marker genes following *MYSM1* depletion, thereby impairing the myogenic development of BSMSCs.

### *MYSM1* promotes the proliferation and differentiation of bovine skeletal muscle cells by regulating *BRG1*

**Figure 4:**
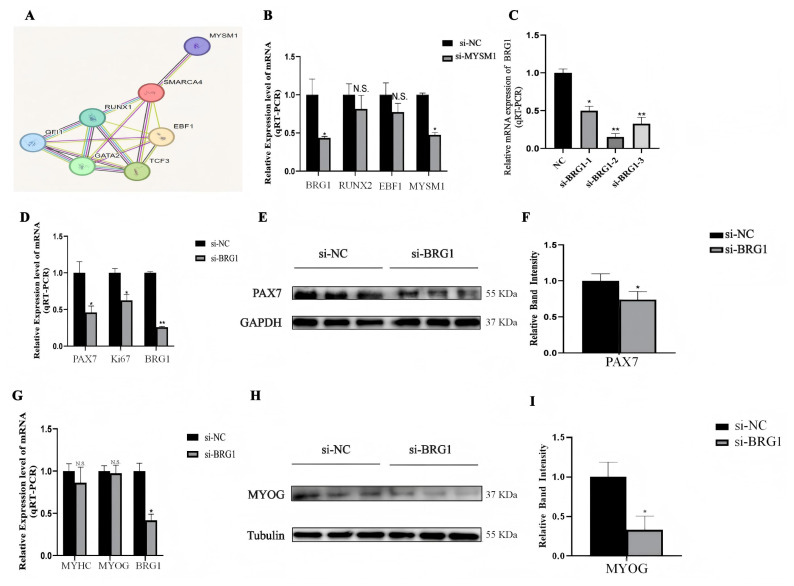
*MYSM1* may promote the growth and differentiation of BSMSCs by regulating *BRG1*. (A) STRING analysis identified proteins that interact with *MYSM1*. (B) Quantitative experiments were conducted to detect changes in the mRNA levels of related interacting proteins following the knocked down of *MYSM1*. (C) qRT-PCR detection of the interference efficiency of *BRG1*. (D, E) Knockdown of *BRG1* inhibited cell proliferation. (D) Knocking down *BRG1* resulted in a decrease in the mRNA levels of the proliferation markers *PAX7* and *Ki67*. (E) The protein expression levels of *PAX7* and *GAPDH* were examined by using Western blot. (F) Then, the band quantification was expressed as the ratio of *PAX7/GAPDH* band intensity. Moreover, knocking down *BRG1* by (G–I) led to a decrease in the mRNA and protein expression levels of the cell differentiation related markers *MYOG* and *MYHC*. Data are presented as mean±SD. * p<0.05, ** p<0.01 vs. control group (one-way ANOVA followed by Dunnett’s test). qRT-PCR, quantitative real-time polymerase chain reaction; BSMSC, bovine skeletal muscle satellite cell; SD, standard deviation; ANOVA, analysis of variance.

To further investigate the regulatory mechanism of *MYSM1*, STRING protein interaction analysis identified the chromatin remodeling factor *BRG1* (*SMARCA4*) as a potential downstream effector of *MYSM1* (Figure 4A). Molecular validation experiments confirmed that *MYSM1* knockdown significantly reduced the mRNA expression level of *BRG1* (p<0.05) (Figure 4B), suggesting that *MYSM1* may maintain *BRG1* expression through transcriptional regulation or mRNA stability mechanisms. Using a specific siRNA-mediated *BRG1* knockdown model, we found that *BRG1* deficiency recapitulated the *MYSM1* knockdown phenotype, characterized by significantly decreased expression of the proliferation markers *PAX7* and *Ki67* (p<0.05), as well as markedly reduced mRNA and protein levels of the key myogenic transcription factor *MYOG* (p<0.05) (Figures 4D–4I). These results indicate that *MYSM1* may act synergistically with *BRG1* to regulate muscle stem cell fate and that loss of *MYSM1* inhibits the growth and differentiation of BSMSCs.

### *MYSM1* promotes the proliferation and differentiation of bovine skeletal muscle cells by regulating *BRG1* to activate the *AKT/mTOR/NF-κB* pathway

**Figure 5:**
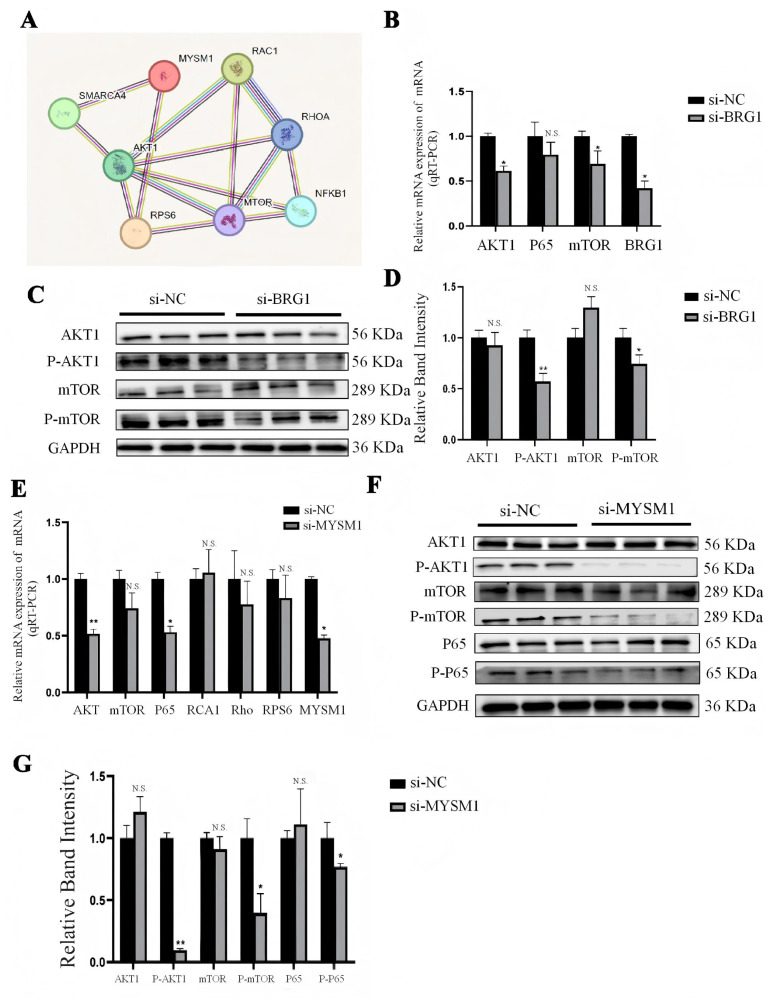
*MYSM1* may promote the proliferation and differentiation of BSMSCs by regulating *BRG1* to activate the *AKT/mTOR/NF-κB* signaling pathway. (A) STRING analysis of the pathway proteins that interact with *MYSM1* and *BRG1*. (B) Quantitative experiments were conducted to detect changes in the mRNA levels of related interaction pathway proteins after knocking down *BRG1*. (C) The expression levels of pathway proteins and the *GAPDH* protein were examined by using Western blot. (D) Then, the band quantification was expressed relative band intensity. (E) Quantitative experiments were conducted to detect changes in the mRNA levels of related interaction pathway proteins after the knockdown of *MYSM1*. (F) The expression levels of pathway proteins and the GAPDH protein were examined by using Western blot. (G) Then, the band quantification was expressed as relative intensity. Data are presented as mean±SD. * p<0.05, ** p<0.01 vs. control group (one-way ANOVA followed by Dunnett’s test). qRT-PCR, quantitative real-time polymerase chain reaction; BSMSC, bovine skeletal muscle satellite cell; SD, standard deviation; ANOVA, analysis of variance.

The STRING protein interaction analysis revealed that *BRG1* and *MYSM1* colocalize as regulatory hub nodes within the *AKT/mTOR/NF-κB* signaling pathway and may interact with key effector molecules such as *NF-κB (p65)*, *AKT*, and *mTOR* (Figure 5A). Consistent with this prediction, *MYSM1* knockdown significantly reduced *BRG1* mRNA expression and decreased the protein levels of the phosphorylated signaling molecules *p-AKT1*, *p-mTOR*, and *p-p65* (p<0.05) (Figures 5B–5G). Furthermore, *BRG1* knockdown reproduced this phenotype, resulting in a significant reduction in *AKT1* mRNA levels and *mTOR* phosphorylation (p<0.05). These findings suggest that *MYSM1* may regulate skeletal muscle development through epigenetic coordination with *BRG1*, thereby modulating the *AKT/mTOR/NF-κB* signaling cascade. This discovery provides a novel theoretical framework for understanding the cross-regulatory mechanisms that link epigenetic regulation with intracellular signaling during muscle development.

## DISCUSSION

This study advances the current understanding of the biological functions of *MYSM1* by extending its known roles beyond the immune system. Previously, *MYSM1* has been recognized for its regulatory functions in promoting B-cell proliferation and differentiation and in maintaining CD8^+^ T-cell homeostasis. In contrast, our findings reveal for the first time that *MYSM1* exhibits a distinct spatiotemporal expression pattern in BSMSCs, characterized by a differentiation-dependent upregulation, with expression levels significantly increased on the second and third days of differentiation (p<0.01).

Functional analyses demonstrated that *MYSM1* knockdown not only significantly suppressed the expression of the proliferation markers *PAX7* and *Ki67*, but also markedly impaired myogenic differentiation, as evidenced by reduced myotube formation and the concomitant downregulation of the key myogenic regulators *MYOG* and *MYHC*. This dual regulatory effect indicates that *MYSM1* serves as a pivotal factor in BSMSCs, orchestrating the dynamic balance between stem cell self-renewal and terminal differentiation.

Histone ubiquitination and deubiquitination function synergistically to modify chromatin structure and regulate gene transcription, often through coordinated interactions with other histone-modifying enzymes. Previous studies examining histone modifications at the *growth factor independence 1* (*GFI1*) locus in HSCs have shown that loss of *MYSM1* alters the local chromatin landscape in HSC progenitors. Specifically, in the *GFI1* enhancer region of *MYSM1*-deficient HSCs, the level of *H3K4me3* is reduced, whereas *H3K27me3* is significantly increased [20], resulting in suppressed HSC proliferation. This observation aligns with the findings of the present study, in which *MYSM1* knockdown similarly decreased *H3K4me3* levels, increased *H3K27me3* levels, and consequently inhibited the proliferation and differentiation of BSMSCs.

In our subsequent investigations, we observed that *MYSM1* knockdown led to a marked reduction in *BRG1* expression. Previous studies examining the role of the chromatin-remodeling enzyme *BRG1* in primary mouse muscle satellite cells have demonstrated that *BRG1* is essential for regulating the transcription factor *PAX7*. Loss of *BRG1* impairs cell proliferation and increases apoptosis. Mechanistically, *BRG1* promotes cell proliferation and survival by facilitating chromatin remodeling and activating transcription at the *PAX7* promoter. Moreover, reintroduction of catalytically active *BRG1* or *PAX7* into *BRG1*-deficient satellite cells rescues the apoptotic phenotype and restores proliferative capacity. These findings indicate that *BRG1* exerts a positive regulatory role in the proliferation and survival of myoblasts [21]. In addition, other studies have shown that *BRG1* enhances chromatin accessibility and promotes de novo RNA synthesis [22]. In addition, this study systematically elucidated the dual regulatory role of *BRG1* in the development of BSMSCs. During the proliferation phase, *BRG1* knockdown significantly reduced the expression of the stem cell maintenance and proliferation marker *PAX7* (p< 0.05), consistent with previous findings in mouse muscle satellite cells [23], thereby confirming that the role of *BRG1* is evolutionarily conserved among mammals. Notably, a unique phenomenon of transcription–translation decoupling was observed in the context of *BRG1*-mediated regulation of myogenic differentiation. Although the mRNA levels of the differentiation-related factors *MYOG* and *MYHC* remained unchanged (p>0.05), their protein levels were significantly decreased (p<0.05). This unexpected result challenges conventional understanding and suggests that BRG1 may regulate protein synthesis through noncanonical mechanisms. One plausible explanation is that *BRG1* may influence the translational efficiency of ribosome-bound mRNAs through chromatin remodeling. Alternatively, *BRG1* may promote the targeted degradation of *MYOG* and *MYHC* proteins by recruiting an E3 ubiquitin ligase complex. Collectively, these findings expand the known functional dimensions of chromatin-remodeling factors and provide new theoretical insights for the development of muscle regeneration strategies that target post-translational regulatory mechanisms.

The growth and development of skeletal muscle are regulated by multiple signaling pathways, among which the *PI3K/AKT* pathway plays a central role in mediating diverse cellular processes, including cell cycle progression, proliferation, and migration, as well as the inhibition of apoptosis. Aberrant activation of this pathway has also been shown to contribute significantly to cancer progression [24]. Previous studies in human colorectal carcinoma cells (HCT116) colorectal cancer cells have shown that suppression of *MYSM1* expression enhances downstream *AKT* activation, thereby promoting signaling events that converge on the *mTOR* pathway and increasing the proliferative and migratory capacities of CRC cells [25]. These findings indicate that, in colorectal cancer cells, *MYSM1* can function as a negative regulator of the *AKT/mTOR* axis [26], whereas our data suggest a positive regulatory role of *MYSM1* on *AKT/mTOR/NF-κB* signaling in BSMSCs, this indicates that *MYSM1* may exert distinct functions across different cellular and biological contexts. Additionally, research on prostate cancer has shown that *MYSM1* interacts with the androgen receptor (AR) and inhibits activation of the *Akt/c-Raf/GSK-3β* signaling cascade [27]. A study investigating the effects of LY294002, an inhibitor of *PI3K*-mediated *AKT* activation, on BSMSCs demonstrated that suppression of *AKT* activity leads to reduced *mTOR* signaling. This inhibition markedly impaired both the proliferation and differentiation of BSMSCs, highlighting the essential regulatory role of the *AKT/mTOR* pathway in skeletal muscle development [28]. In addition, both *in vitro* and *in vivo* studies have demonstrated that *Prostaglandin-endoperoxide synthase 1* (*PTGS1)* is indispensable for the osteogenic differentiation of adipose-derived stem cells (ASCs), acting through modulation of the *NF-κB* signaling pathway to regulate their osteogenic potential [29]. Another study reported, for the first time, that tumor necrosis *factor superfamily member 14* (*TNFSF14*) inhibits melanin synthesis in primary cultured human epidermal melanocytes by activating the *NF-κB* signaling pathway in B cells; inhibition of this pathway effectively blocks *TNFSF14*-induced depigmentation [30].

In the present study, *MYSM1* knockdown resulted in a marked reduction in *BRG1* mRNA expression and induced a cascade of signaling dysregulation, characterized by significant decreases in the phosphorylated forms of key proteins—*p-AKT1, p-mTOR,* and *p-p65*. Further investigation revealed that *BRG1* knockdown recapitulated this phenotype, establishing a three-tier regulatory cascade of “*MYSM1* → *BRG1* → *AKT/mTOR/NF-κB*.” This cross-hierarchical regulatory network integrates three molecular layers: epigenetic modification (*MYSM1*-mediated deubiquitination of *H2AK119ub1*), chromatin remodeling (*BRG1*-driven modulation of DNA accessibility), and intracellular signal transduction (phosphorylation cascade). Together, these interactions maintain *AKT/mTOR* pathway activity to regulate cell cycle progression and modulate *NF-κB* nuclear translocation to activate proliferation-associated gene transcription, thereby determining the fate of BSMSCs.

This discovery establishes an innovative theoretical framework for elucidating the epigenetic–signaling cross-regulation underlying muscle development and provides a mechanistic basis for accelerating genetic improvement and enhancing meat quality in high-performance beef cattle breeding programs.

## CONCLUSION

**Figure 6:**
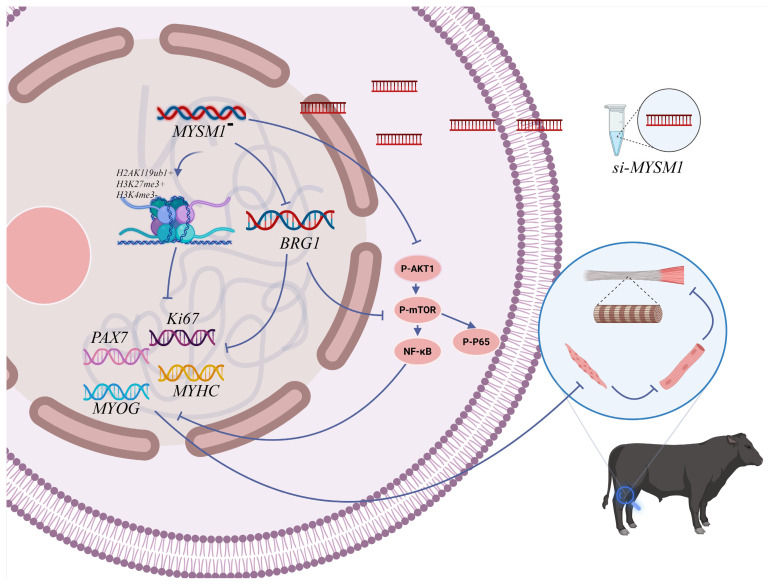
*MYSM1* regulates the proliferation and differentiation mechanism of bovine skeletal muscle satellite cells. Knocking down *MYSM1* reduces *BRG1* expression and activates the *AKT/mTOR/NF-κB* signaling pathway, thereby inhibiting the proliferation and differentiation of bovine skeletal muscle satellite cells. Created with BioRender.com.

This study revealed that the histone deubiquitinase *MYSM1* regulates *BRG1* expression and modulates the activity of the *AKT/mTOR/NF-κB* signaling pathway, thereby establishing a three-tier regulatory network integrating epigenetic modification, chromatin remodeling, and signal transduction. Through this “epigenetic–chromatin remodeling–signaling” cascade, *MYSM1* orchestrates the proliferation and differentiation of BSMSCs. These findings elucidate the molecular mechanism by which *MYSM1* contributes to muscle development and provide a dual-dimensional theoretical basis for advancing molecular breeding and muscle regeneration therapies in beef cattle (Figure 6).

## Figures and Tables

**Table 1:** Sequence information

Fragment name	Sequence
si-bta-*MYSM1*_001	CAACCTGAATGCTGTGAAA
si-bta-*MYSM1*_002	CACCAGAACAAGAAATAGA
si-bta-*MYSM1*_003	GGAATGGAATAACAGATGA

**Table 2:** Sequence information

Fragment name	Sequence
si-bta-*BRG1*_001	GCCAATGGAGTCCATGCAT
si-bta-*BRG1*_002	CGCAGATCATGGCCTACAA
si-bta-*BRG1*_003	GCATCGCACACCGAATTCA

**Table 3:** qRT-PCR primer sequences

Gene ID	Primer sequence (5–3)	Produc length
*MYSM1*	F: AGAGTCTGCAGAAAACAGCA R: CCAGCCCTTGTTCAAACAGC	108
*GAPDH*	F: TGTTGTGGATCTGACCTGCC R: AAGTCGCAGGAGACAACCTG	135
*Pax7*	F: AGCCAGAGTTTCAACGGGAG R: GTCGCCAACAGACAACACAC	93
*PCNA*	F: AAGCCACTCCACTGTCTCCT R: CATCCTCGATCTTGGGAGCC	123
*MYHC*	F: TGCTCATCTCACCAAGTTCC R: CACTCTTCACTCTCATGGAC	150
*MYOG*	F: CAAATCCACTCCCTGAAA R: GCATAGGAAGAGATGAACA	93
*BRG1*	F: CGGCTGCTTCTTTGTTTCGT R: GGTCCGGTGTGGACATCTTC	155
*EBF1*	F: TCGGAAGGTACGCCCTCTTA R: CAGACCAGCATGGTACCGAA	155
*RUNX1*	F: TGTGAAAACTTCTTTGGGCTC R: TACTCTGCAAAGCCCTGTGG	142
*AKT1*	F: TTACCTGCACTCGGAAAAGGAA R: AGTCCGAAGTCGGTGATCTTG	99
*NF-κB*	F: ACCAAGACCCACCCCACTAT R: CTCATAGAAGCCATCCCGGC	141
*mTOR*	F: GGTTTTGGAACGAAACCCCG R: CCATGAGGCCTTGGTGAGAG	118

qRT-PCR, quantitative real-time polymerase chain reaction.

## Data Availability

Upon reasonable request, the datasets of this study can be available from the corresponding author.
